# Doping dependence of low-energy quasiparticle excitations in superconducting Bi2212

**DOI:** 10.1186/1556-276X-8-515

**Published:** 2013-12-05

**Authors:** Akihiro Ino, Hiroaki Anzai, Masashi Arita, Hirofumi Namatame, Masaki Taniguchi, Motoyuki Ishikado, Kazuhiro Fujita, Shigeyuki Ishida, Shinichi Uchida

**Affiliations:** 1 Graduate School of Science, Hiroshima University, Higashi-Hiroshima 739-8526, Japan; 2 Graduate School of Engineering, Osaka Prefecture University, Sakai 599-8531, Japan; 3 Hiroshima Synchrotron Radiation Center, Hiroshima University, Higashi-Hiroshima 739-0046, Japan; 4 Department of Physics, University of Tokyo, Tokyo 113-0033, Japan; 5 National Institute of Advanced Industrial Science and Technology, Tsukuba 305-8568, Japan; 6 Research Center for Neutron Science and Technology, CROSS, Tokai, Ibaraki 319-1106, Japan; 7 Laboratory for Atomic and Solid State Physics, Department of Physics, Cornell University, Ithaca, New York 14853, USA

**Keywords:** High-*T*_c_ cuprate, Bi2212, ARPES, Superconducting gap, Effective mass, Coupling strength, 74.25.Jb, 74.72.-h, 79.60.i

## Abstract

The doping-dependent evolution of the *d*-wave superconducting state is studied from the perspective of the angle-resolved photoemission spectra of a high-*T*_c_ cuprate, Bi_2_Sr_2_CaCu_2_ O_8+*δ*_ (Bi2212). The anisotropic evolution of the energy gap for Bogoliubov quasiparticles is parametrized by critical temperature and superfluid density. The renormalization of nodal quasiparticles is evaluated in terms of mass enhancement spectra. These quantities shed light on the strong coupling nature of electron pairing and the impact of forward elastic or inelastic scatterings. We suggest that the quasiparticle excitations in the superconducting cuprates are profoundly affected by doping-dependent screening.

## Background

Electronic excitations dressed by the interaction with the medium are called quasiparticles. They serve as a direct probe of the anisotropic order parameter of a superconducting phase and also as a clue to the electron-pairing glue responsible for the superconductivity. In fact, the major unresolved issues on the mechanism of high-*T*_c_ superconductivity depend on the low-energy quasiparticle excitations. The superconducting order parameter, which is typified by the particle-hole mixing and gives rise to Bogoliubov quasiparticles (BQPs), manifests itself as an energy gap in quasiparticle excitation spectra. In cuprate superconductors, however, the energy gap increases against the decrease in critical temperature *T*_c_ with underdoping and is open even at some temperatures above *T*_c_[1-3]. In the direction where the *d*-wave order parameter disappears, renormalization features have been extracted quantitatively from the gapless continuous dispersion of nodal quasiparticles (NQPs), suggesting strong coupling with some collective modes [4]. Nevertheless, the origins of these features remain controversial [4,5].

In this paper, we address the doping dependence of BQP and NQP of a high-*T*_c_ cuprate superconductor, Bi_2_Sr_2_CaCu_2_O_8+*δ*_ (Bi2212), on the basis of our recent angle-resolved photoemission (ARPES) data [6-8]. The use of low-energy synchrotron radiation brought about improvement in energy and momentum resolution and allowed us to optimize the excitation photon energy. After a brief description of BQP and NQP spectral functions, we survey the superconducting gap anisotropy on BQPs and the renormalization features in NQPs. In light of them, we discuss possible effects of doping-dependent electronic screening on the BQP, NQP, and high-*T*_c_ superconductivity.

## Methods

High-quality single crystals of Bi2212 were prepared by a traveling-solvent floating-zone method, and hole concentration was regulated by a post-annealing procedure. In this paper, the samples are labeled by the *T*_c_ value in kelvin, together with the doping-level prefix, i.e. underdoped (UD), optimally doped (OP), or overdoped (OD). ARPES experiments were performed at HiSOR BL9A in Hiroshima Synchrotron Radiation Center. The ARPES data presented here were taken with excitation-photon energies of *hν* = 8.5 and 8.1 eV for the BQP and NQP studies, respectively, and at a low temperature of *T* = 9 - 10 K in the superconducting state. Further details of the experiments have been described elsewhere [7-9].

The relation between a bare electron and a renormalized quasiparticle is described in terms of self-energy Σ_**k**_(*t*), which can be regarded as a factor of feedback on the wave function from past to present through the surrounding medium. Incorporating a feedback term into the Schrödinger equation, we obtain

(1) $$\frac{d\psi_{k}(t)}{\text{dt}}=-i\omega_{k}^{0}\psi_{k}(t)-i\int_{-\infty }^{t}\Sigma_{k}(t-t^{\prime })\psi_{k}(t^{\prime })dt^{\prime },$$

where *ψ*_**k**_(*t*) and $\omega_{k}^{0}$ denote a wave function and a bare-electron energy, respectively. It is obvious from Equation 1 that the self-energy is a linear response function. Therefore, its frequency representation, Σ_**k**_(*ω*), obeys the Kramers-Kronig relation. As the solution of Equation 1, we obtain the form of dressed Green’s function,

(2) $$G_{k}(\omega )=\frac{1}{\omega -\omega_{k}^{0}-\Sigma_{k}(\omega )}.$$

The spectral function given by *A*_**k**_(*ω*) = - Im *G*_**k**_(*ω*)/*π* is directly observed by ARPES experiments. The extensive treatments of the ARPES data in terms of Green’s function are given elsewhere [10].

## Results

### Superconducting gap anisotropy

In the superconducting state, the condensate of electron pairs allows the particle-like and hole-like excitations to turn into each other. Hence, the wave function of a hole-like excitation also comes back by way of the particle-like excitation. Such a feedback has a sign-reversed eigenenergy, $-\omega_{k}^{0}$, and is expressed by $\Sigma_{k}(t)=-i\Theta (t)\Delta_{k}^{2}e^{i\omega_{k}^{0}t}e^{-\gamma_{k}t}-i\Gamma_{k}\delta (t)$, where Θ(*t*), Δ_**k**_, *γ*_**k**_ and Γ_**k**_ denote the step function, the particle-hole off-diagonal element, and the scattering rates of the intermediate and bare-particle states, respectively. The Fourier transform of Σ_**k**_(*t*) gives the frequency representation of the self-energy of the BQPs,

(3) $$\Sigma_{k}(\omega )=\frac{\Delta_{k}^{2}}{\omega +\omega_{k}^{0}+i\gamma_{k}}-i\Gamma_{k}.$$

**Figure 1:**
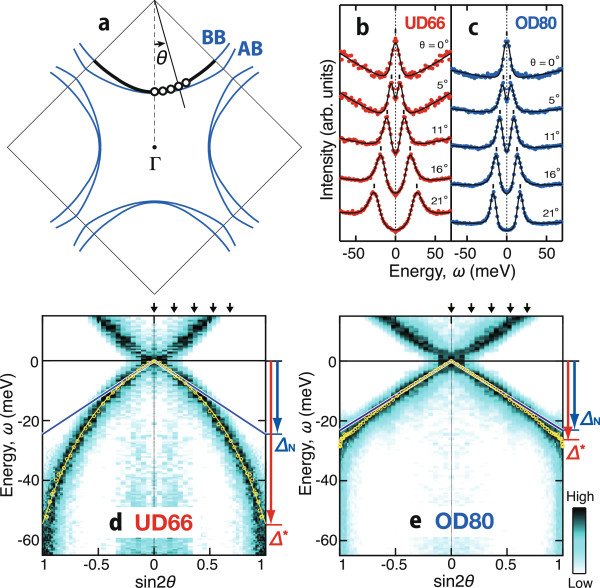
**Superconducting gap manifested in BQP spectra.** The data are for underdoped and overdoped Bi2212 samples with *T*_c_ = 66 and 80 K (UD66 and OD80, respectively) [8]. **(a)** Momentum-space diagram for an off-node angle, *θ*, and a bonding-band (BB) Fermi surface along which the ARPES spectra were taken. **(b, c)** Symmetrized energy distribution curves (colored circles) and their fits (black curves). **(d, e)** ARPES spectral images as a function of energy *ω* and sin 2*θ*. Superimposed are the gap energies (yellow circles) and high-harmonic fit (yellow curve) as functions of sin 2*θ*.

Figure 1 shows the ARPES spectra of BQPs for underdoped and overdoped Bi2212 samples with *T*_c_ = 66 and 80 K (UD66 and OD80, respectively) [8]. As shown in Figure 1b,c, an energy distribution curve was extracted from the minimum gap locus for each off-node angle *θ* and symmetrized with respect to the Fermi energy *ω* = 0. These spectra were well fitted with a phenomenological function,

(4) $$A(\omega )=-\frac{1}{\pi }\text{Im}\left(\frac{1}{\omega +i\Gamma -\Delta^{2}/\omega }\right),$$

except for a featureless background. Equation 4 is deduced from Equation 3 and $\omega_{k}^{0}=0$, neglecting *γ*_**k**_ after Norman et al. [11]. Figure 1b,c exemplifies that the superconducting gap energy Δ at each *θ* is definitely determined by sharp spectral peaks. In Figure 1d,e, the obtained gap energies (small yellow circles) are plotted over the image of spectral intensity as a function of sin 2*θ*, so that the deviation from a *d*-wave gap is readily seen with reference to a straight line. While the superconducting gap of the overdoped sample almost follows the *d*-wave line, that of the underdoped sample is deeply curved against sin 2*θ*. Furthermore, Figure 1d indicates that the deviation from the *d*-wave gap penetrates into the close vicinity of the node and that it is difficult to define the pure *d*-wave region near the node. Therefore, the next-order harmonic term, sin 6*θ*, has been introduced, so that the smooth experimental gap profile is properly parametrized [12-14]. The next-order high-harmonic function is also expressed as Δ(*θ*) = Δ_N_ sin 2*θ* + (Δ^∗^-Δ_N_)(3 sin 2*θ*- sin 6*θ*)/4, where the antinodal and nodal gap energies are defined as Δ^∗^ = Δ(*θ*)|_*θ*=45°_ and $\Delta_{N}={\left(\frac{1}{2}(d\Delta /d\theta )\right|}_{\theta =0}$, respectively, so that Δ_N_/Δ^∗^ = 1 is satisfied for a pure *d*-wave gap.

**Figure 2:**
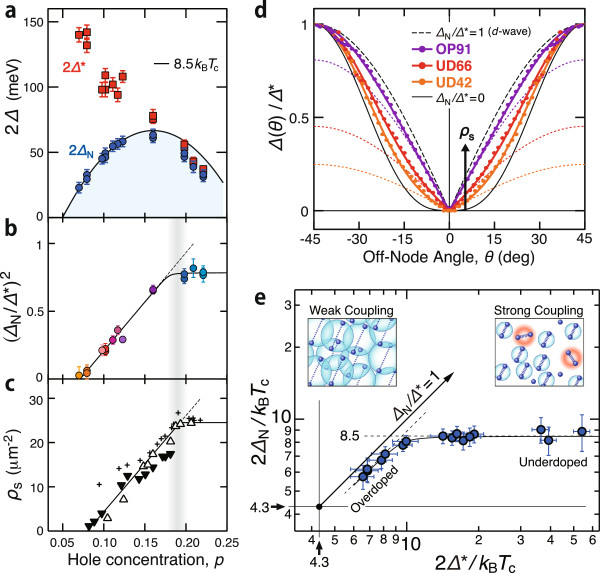
**Doping dependences of superconducting gap parameters.** **(a)** Nodal gap energy 2Δ_N_ (blue circles) and antinodal gap energy 2Δ^∗^ (red squares) [8]. The solid curve denotes an energy of 8.5*k*_B_*T*_c_. **(b)** Square of nodal-to-antinodal gap ratio (Δ_N_/Δ^∗^)^2^ determined from ARPES [8]. **(c)** Superfluid density *ρ*_s_ determined from magnetic penetration depth (triangles) [21] and from heat capacity (crosses) [22]. **(d)** Superconducting gap profiles normalized to the antinodal gap for underdoped and optimally doped samples with *T*_c_ = 42, 66, and 91 K (UD42, UD66, and OP91, respectively). **(e)** Correlation between 2Δ_N_/*k*_B_*T*_c_ and 2Δ^∗^/*k*_B_*T*_c_ ratios. The insets illustrate the occurrence of incoherent electron pairs in strong coupling superconductivity.

The doping dependences of the superconducting gap parameters are summarized in Figure 2. One can see from Figure 2a that as hole concentration decreases with underdoping, the nodal gap energy 2Δ_N_ closely follows the downward curve of 8.5*k*_B_*T*_c_ in contrast to the monotonic increase in the antinodal gap energy 2Δ^∗^. It seems reasonable that *T*_c_ primarily depends on Δ_N_ rather than Δ^∗^ for the underdoped Bi2212, because it follows from 2Δ^∗^ ≫ 4*k*_B_*T*_c_ that the thermal quasiparticle excitations concentrate in the vicinity of the node and hardly occur around the antinode. The relevance of the nodal excitations has also been suggested by various experiments [15-19]. Then, the problem with *T*_c_ is that the nodal gap Δ_N_ is suppressed relative to the antinodal gap Δ^∗^. This behavior can be associated with low superfluid density *ρ*_s_[20]. Figure 2b,c shows that the doping dependence of the nodal-to-antinodal gap ratio Δ_N_/Δ^∗^ is quite similar to that of the square-root superfluid density $\sqrt{\rho_{s}}$[8,21,22]. The normalized gap plot in Figure 2d indicates that what occurs with underdoping is analogous to the nodal gap suppression observed with increasing temperature [17] in terms of the decrease in *ρ*_s_. It is notable that the square-root dependence on *ρ*_s_ is a typical behavior of the order parameter as expected from the Ginzburg-Landau theory [23]. These findings can be written down in a simple relational formula,

(5) $$8.5k_{B}T_{c}=2\Delta_{N}=\Delta^{*}\;\sqrt{\frac{\rho_{s}}{\rho_{s}^{0}}},$$

where $\rho_{s}^{0}≃31\mu m^{-2}$, for a wide hole-concentration range of Bi2212.

As presented in Figure 2e, the correlation between the nodal and antinodal gaps provides a perspective of crossover for our empirical formula (Equation 5). It is deduced from the conventional Bardeen-Cooper-Schrieffer (BCS) theory that 2Δ/*k*_B_*T*_c_ = 4.3 in the weak coupling limit for the *d*-wave superconducting gap [23]. However, the critical temperature *T*_c_ is often lower than that expected from the weak coupling constant and a given Δ as an effect of strong coupling. Thus, the gap-to- *T*_c_ ratio is widely regarded as an indicator for the coupling strength of electron pairing and adopted for the coordinate axes in Figure 2e. As hole concentration decreases from overdoped to underdoped Bi2212, the experimental data point moves apart from the weak coupling point toward the strong coupling side, and a crossover occurs at 8.5, which is about twice the weak coupling constant. It appears that the evolution of Δ_N_ is confined by two lines as Δ_N_ ≤ 0.87Δ^∗^ and 2Δ_N_ ≤ 8.5*k*_B_*T*_c_. As illustrated in the insets of Figure 2e, the strong coupling allows the electrons to remain paired with incoherent excitations. As a result, the superconducting order parameter is reduced with respect to the pairing energy. Indeed, it has been shown that the reduction factor due to the incoherent pair excitations has a simple theoretical expression $\sqrt{\rho_{s}/\rho_{s}^{\text{BCS}}}$ and that the nodal and antinodal spectra are peaked at the order parameter and at the pairing energy, respectively, taking into account a realistic lifetime effect [24,25]. Therefore, the latter part of Equation 5 is consistent with the strong coupling scenario, and furthermore, the two distinct lines in Figure 2e are naturally interpreted as the energies of the condensation and formation of the electron pairs.

### Renormalization features in dispersion

**Figure 3:**
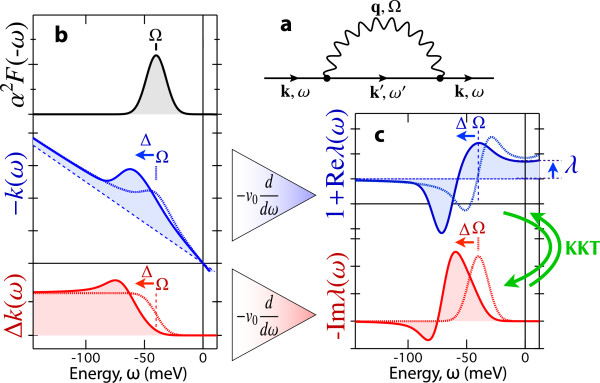
**Simulation for a single coupling mode at Ω = 40 meV.** Dotted and solid curves denote those with and without a *d*-wave gap of Δ = 30 meV, respectively. **(a)** Diagram of electron-boson interaction. **(b)** Eliashberg coupling function *α*^2^*F*(-*ω*), dispersion *k*(*ω*) = [*ω* + ReΣ(*ω*)]/*v*_0_, and momentum width Δ*k*(*ω*) = -ImΣ(*ω*)/*v*_0_. **(c)** Real and imaginary parts of 1 + *λ*(*ω*).

In the nodal direction where the order parameter disappears, one can investigate the fine renormalization features in dispersion. They reflect the intermediate-state energy in coupling between an electron and other excitations, and thus provide important clues to the pairing interaction. As for the electron-boson coupling, the intermediate state consists of a dressed electronic excitation and an additional bosonic excitation (Figure 3a). Averaging the momentum dependence for simplicity, the energy distribution of the intermediate state is expressed by *A*(*ω* - Ω) Θ(*ω* - Ω)+*A*(*ω* + Ω) Θ(-*ω* - Ω) for a given boson energy Ω and for zero temperature, owing to the Pauli exclusion principle. Therefore, taking into account the effective energy distribution of the coupled boson, *α*^2^*F*(Ω), the self-energy is written down as follows:

(6) $$\begin{array}{ll} \;\Sigma (t) & =-i\;\int \;d\omega^{\prime }\;\Theta (t)\;e^{-i\omega^{\prime }t}\;\int_{0}^{\infty }\;d\Omega \;\alpha^{2}F(\Omega )\\ & \;\times \left[A(\omega^{\prime }\;-\;\Omega )\;\Theta (\omega^{\prime }\;-\;\Omega )\;+\;A(\omega^{\prime }\;+\;\Omega )\;\Theta (-\omega^{\prime }\;-\;\Omega )\right], \end{array}$$

(7) $$\begin{array}{ll} \;\Sigma (\omega ) & =\int \frac{d\omega^{\prime }}{\omega -\omega^{\prime }+i0^{+}}\int_{0}^{\infty }\;d\Omega \;\alpha^{2\;}F(\Omega )\\ & \;\times \left[A(\omega^{\prime }\;-\;\Omega )\;\Theta (\omega^{\prime }\;-\;\Omega )\;+\;A(\omega^{\prime }\;+\;\Omega )\;\Theta (-\omega^{\prime }\;-\;\Omega )\right], \end{array}$$

where 0^+^ denotes a positive infinitesimal.

In ARPES spectra, the real and imaginary parts of self-energy manifest themselves as the shift and width of spectral peak, respectively. Specifically, provided that the momentum dependence of Σ_**k**_(*ω*) along the cut is negligible, and introducing bare electron velocity *v*_0_ by $\omega_{k}^{0}=v_{0}k$, it follows from Equation 2 that the momentum distribution curve for a given quasiparticle energy *ω* is peaked at *k*(*ω*) = [*ω*-ReΣ(*ω*)]/*v*_0_ and has a natural half width of Δ*k*(*ω*) = - ImΣ(*ω*)/*v*_0_.

We argue that the mass enhancement function defined as the energy derivative of the self-energy, *λ*(*ω*) ≡ -(*d*/*dω*)Σ(*ω*), is useful for the analysis of NQP [7,26]. The real and imaginary parts of *λ*(*ω*) are directly obtained from the ARPES data as the inverse of group velocity, *v*_g_(*ω*), and as the differential scattering rate, respectively.

(8) $$\begin{array}{l} \;\frac{d}{d\omega }k(\omega )=\frac{1}{v_{0}}\left[1+\text{Re}\lambda (\omega )\right]=\frac{1}{v_{g}(\omega )} \end{array}$$

(9) $$\begin{array}{l} \frac{d}{d\omega }\Delta k(\omega )=\frac{1}{v_{0}}\text{Im}\lambda (\omega ) \end{array}$$

We note that -Im*λ*(*ω*) represents the energy distribution of the impact of coupling with other excitations and can be taken as a kind of coupling spectrum. However, it should be emphasized that -Im*λ*(*ω*) is expressed as a function of quasiparticle energy *ω*, whereas the widely used Eliashberg coupling function *α*^2^*F*(Ω) is expressed as a function of boson energy Ω. For example, a simulation of *λ*(*ω*) using Equations 7 to 9 is presented in Figure 3b,c, where a single coupling mode is given at Ω = 40 meV. One can see that the peak of *α*^2^*F*(-*ω*) is reproduced by -Im*λ*(*ω*), provided that *A*(*ω*) is gapless and approximated by a constant. As an energy gap of Δ opens in *A*(*ω*), the peak in -Im*λ*(*ω*) is shifted from Ω into Ω + Δ. Nevertheless, irrespective of *A*(*ω*), the causality of Σ(*ω*) is inherited by *λ*(*ω*), so that Re*λ*(*ω*) and Im*λ*(*ω*) are mutually convertible through the Kramers-Kronig transform (KKT). The directness and causality of *λ*(*ω*) enable us to decompose the quasiparticle effective mass without tackling the integral inversion problem in Equation 7.

**Figure 4:**
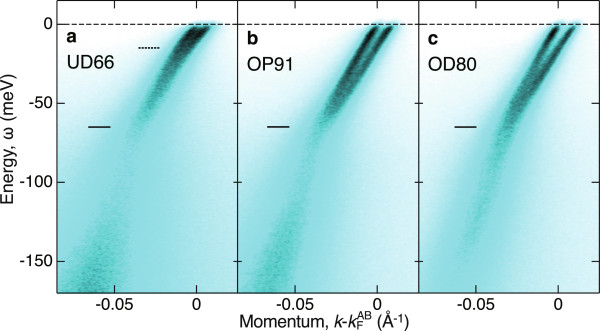
**Dispersion kinks manifested in NQP spectra.** The ARPES spectra were taken in the superconducting state for Bi2212 [7]. **(a)** Underdoped sample with *T*_c_ = 66 K (UD66). **(b)** Optimally doped sample with *T*_c_ = 91 K (OP91). **(c)** Overdoped sample with *T*_c_ = 80 K (OD80).

Figure 4 shows the ARPES spectra along the nodal cut perpendicular to the Fermi surface for the superconducting Bi2212 [7]. Although the splitting due to the CuO_2_ bilayer is minimum at the nodes, it has clearly been observed by using some specific low-energy photons [6-8]. A prominent kink in the NQP dispersion is observed at 65 meV for all the doping level, as has been reported since early years [4]. In addition to this, another small kink at 15 meV is discernible in the raw spectral image of the underdoped sample (UD66) [7,27].

**Figure 5:**
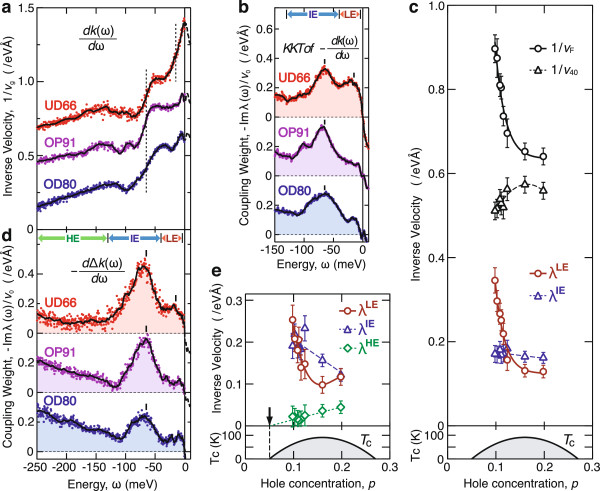
**Doping dependences of NQP properties.** The real and imaginary parts of mass enhancement spectra were directly deduced from the APRES data shown in Figure 4[7]. **(a)** Inverse group velocity, 1/*v*_g_(*ω*) = [1 + Re *λ*(*ω*)]/*v*_0_, determined from (*d*/*dω*) *k*(*ω*). **(b)** Differential scattering rate -Im *λ*(*ω*)/*v*_0_, deduced from the Kramers-Kronig transform (KKT) of **(a)**. **(c)** Partial coupling constants, *λ*^LE^ (red circles) and *λ*^IE^ (blue triangles), deduced from **(b)**. Also shown are the inverse group velocities at *ω* = 0 (black circles) and at *ω* = -40 meV (black triangles). **(d)** Differential scattering rate -Im *λ*(*ω*)/*v*_0_, directly determined from -(*d*/*dω*) Δ*k*(*ω*). **(e)** Partial coupling constants, *λ*^LE^ (red circles), *λ*^IE^ (blue triangles), and *λ*^HE^ (green diamonds), deduced from **(d)**.

The fine renormalization features in the NQP dispersion were determined by fitting the momentum distribution curves with double Lorentzian. Figure 5a,d shows the real and imaginary parts of *λ*(*ω*)/*v*_0_ experimentally obtained as the energy derivatives of the peak position and width, respectively. The KKT of Re*λ*(*ω*)/*v*_0_ in Figure 5a is shown in Figure 5b as Im*λ*(*ω*)/*v*_0_, which is comparable with the data in Figure 5d. A step-like mass enhancement in Figure 5a and a peak-like coupling weight in Figure 5b,d are consistently observed at 65 meV. This is a typical behavior of the mode coupling, as shown by the simulation in Figure 3. It is also found that an additional feature around 15 meV is dramatically enhanced with underdoping. In order to deduce the partial coupling constant, we express the mass enhancement factor *λ* as the form of KKT,

(10) $$\lambda =\text{Re}\lambda (0)=\frac{2}{\pi }\int_{0}^{\infty }\frac{\text{Im}\lambda (\omega )}{\omega }d\omega .$$

Dividing the energy range of the integral in Equation 10, one can quantify the contribution from a particular energy part. We refer to the KKT integrals of Im *λ*(*ω*)/*v*_0_ for the low-energy (LE; 4 < |*ω*| < 40 meV), intermediate-energy (IE; 40 < |*ω*| < 130 meV), and high-energy (HE; 130 < |*ω*| < 250 meV) parts as *λ*^LE^/*v*_0_ (red circles), *λ*^IE^/*v*_0_ (blue triangles), and *λ*^HE^/*v*_0_ (green diamonds), respectively. Those obtained from the data in Figure 5b,d are plotted in Figure 5c,e, respectively. Also shown in Figure 5c are the inverse group velocities at *ω* = 0 meV (black circles) and at *ω* = -40 meV (black triangles). Figure 5c and Figure 5e consistently indicate that as hole concentration decreases, the contribution of the low-energy part rapidly increases and becomes dominant over the other parts.

Possible origins of the low-energy kink are considered from the energy of 15 meV and the evolution with underdoping. The quasiparticles that can be involved in the intermediate states are limited within the energy range of |*ω*| ≤ 15 meV, and the irrelevance of the antinodal states is deduced from the simulation in Figure 3c. Therefore, the low-energy kink is due to the near-nodal scatterings with small momentum transfer. The candidates for bosonic forward scatterers are the low-frequency phonons, such as the acoustic phonons and the *c*-axis optical phonons involving heavy cations [7,28-31]. On the other hand, it has also been argued that the elastic forward scattering by off-plane impurities may give rise to the low-energy kink for the *d*-wave superconductors [7,32]. In usual metal, both the potentials of the low-frequency phonons and the static impurities are strongly screened by the rapid response of electronic excitations. Therefore, the enhancement of the low-energy kink suggests the breakdown of electronic screening at low hole concentrations [7,28].

The dispersion kink at 65 meV has been ascribed to an intermediate state consisting of an antinodal quasiparticle and the *B*_1g_ buckling phonon of Ω ∼ 35 meV [33]. However, the mass enhancement spectra in Figure 5a,b,d are suggestive of the presence of multiple components in the intermediate-energy range.

## Discussion

We found that both the superconducting gap anisotropy and the renormalized dispersion show the striking evolution with underdoping. These behaviors are considered to be dependent on the extent of the screening. In association with the forward elastic or inelastic scatterings, the screening breakdown would enhance the low-energy kink. From the aspect of the impact of off-plane impurities, the inadequacy of static screening would inevitably lead to the nanoscale inhomogeneities, as observed by scanning tunneling microscopy experiments [34]. The forward scatterings by the remaining potential would generate additional incoherent pair excitations, as expected from the nodal gap suppression at low temperatures [8,25]. From the aspect of the electron-phonon coupling, the inhomogeneous depletion of the electrons for screening may considerably increase the coupling strength, providing an account for the unexpectedly strong dispersion kink [35] and a candidate for the strong pairing interaction [8]. The former and latter aspects have negative and positive effects, respectively, on the superconductivity. Thus, we speculate that the doping dependence of *T*_c_ is eventually determined by the balance between these effects.

## Conclusions

Summarizing, the evolution of a *d*-wave high-*T*_c_ superconducting state with hole concentration has been depicted on the basis of the high-resolution ARPES spectra of the quasiparticles and discussed in relation to the screening by electronic excitations. The divergence between the nodal and antinodal gaps can be interpreted as an effect of the incoherent pair excitations inherent in the strong coupling superconductivity. The low-energy kink, which rapidly increases with underdoping, should be caused by the forward elastic or inelastic scatterings, although it remains as an open question which scattering is more dominant. The quantitative simulation of the doping-dependent effect will be helpful for resolving this problem.

## Abbreviations

AB: Antibonding band; ARPES: Angle-resolved photoemission spectroscopy; BB: Bonding band; BCS: Bardeen-Cooper-Schrieffer; Bi2212: Bi_2_Sr_2_CaCu_2_ O_8+*δ*_; BQP: Bogoliubov quasiparticle; HE: High energy; IE: Intermediate energy; KKT: Kramers-Kronig transform; LE: Low energy; NQP: Nodal quasiparticle; OD: Overdoped; OP: Optimally doped; UD: Underdoped.

## Competing interests

The authors declare that they have no competing interests.

## Authors’ contributions

AI wrote the manuscript. HA and AI designed the experiment and analyzed the data with support from MT. HA acquired the ARPES data with support from AI, MA, and HN. High-quality single-crystalline samples were grown by MI, KF, SI, and SU. All authors discussed the results and commented on the manuscript. All authors read and approved the final manuscript.
